# Clinicopathological Study on Morphological Subtypes of Hepatocellular Carcinoma: A Single Tertiary Referral Center Experience

**DOI:** 10.1002/cnr2.70127

**Published:** 2025-02-14

**Authors:** C. H. A. Saler, S. Shuai, J. C. Beckervordersandforth, D. Rennspiess, G. Roemen, T. Gevers, M. C. F. Stoehr‐Kleinegris, S. A. W. Bouwense, M. J. L. Dewulf, M. M. E. Coolsen, M. H. A. Bemelmans, S. W. Olde Damink, V. Winnepenninckx, A. zur Hausen, M. Kramer, I. V. Samarska

**Affiliations:** ^1^ Department of Pathology GROW‐School for Oncology and Reproduction, Maastricht University Medical Center + Maastricht the Netherlands; ^2^ Department of Internal Medicine GROW‐School for Oncology and Reproduction, Maastricht University Medical Center + Maastricht the Netherlands; ^3^ Department of Surgery School of Nutrition and Translational Research in Metabolism (NUTRIM), School of Nutrition and Translational Research in Metabolism, Maastricht University Maastricht the Netherlands

**Keywords:** cancer care, liver cancer, pathology, tumor heterogeneity

## Abstract

**Aim:**

We aimed to analyze hepatocellular carcinoma (HCC) morphological subtypes characterized according to the WHO classification and the International Collaboration on Cancer Reporting (ICCR) recommendations, and their prognostic features in a Dutch population.

**Methods and Results:**

This retrospective study in a tertiary referral center included the histopathological revision of 62 HCC resection specimens, obtained from 22 female and 40 male patients (median age: 67 years), in a period between 2011 and 2021 at the Maastricht University Medical Center +. Clinical data, morphological subtypes, growth pattern (GP), tumor grade, tumor extension, margins, and vascular and perineural invasion were collected. Eighteen cases were assigned a specific morphologic subtype and steatohepatic HCC was the most common in our cohort. Twenty‐one tumors classified as conventional type HCC (HCC‐NOS), commonly exhibiting two concurrent GPs. Twenty‐three cases revealed a heterogeneous morphologic differentiation, compromising the combination of HCC‐NOS with another morphologic subtype, most frequently a steatohepatitic component. Comparison of HCC‐NOS and HCC with heterogeneous morphology did not show significant differences in the main clinicopathological characteristics and survival.

**Conclusion:**

Although the most common morphologic subtype was steatohepatitic HCC, the majority of cases demonstrated multiple morphologic patterns. In case of HCC‐NOS, heterogeneous GPs were often observed. Therefore, a histomorphological diagnosis based on a single tumor biopsy specimen may lead to incorrect classification of HCC. Sufficient tumor sampling of HCC resection specimens is required for the complete evaluation of all histomorphological features followed by correct subclassification in order to meet the clinical needs regarding prognostic relevance and patient follow‐up.

## 1 Introduction

Hepatocellular carcinoma (HCC) is the most common primary liver cancer and the fifth most common cancer worldwide [1, 2]. Both, the prevalence and incidence of HCC are rising [3]. HCCs are a group of tumors with diverse histopathological morphology [2, 4, 5, 6, 7, 8, 9]. In 2019, the World Health Organization (WHO) updated the histopathological classification of HCC, recognizing 12 distinct morphologic subtypes and six provisional subtypes, which have different implications on prognosis [1, 10, 11]. According to this classification, approximately 35% of HCCs exhibit a specific morphological subtype [11, 12]. For example, clear‐cell hepatocellular carcinoma (CC‐HCC), defined by clear‐cell morphology similar to other clear‐cell carcinomas, is thought to present less commonly with vascular invasion and overall has a better prognosis [11]. Steatohepatitic HCC (S‐HCC) has a distinct morphology, defined by macrovesicular steatosis, balloon cells, and Mallory‐Denk bodies [13]. Its prognosis is similar to conventional type HCC (HCC, Not Otherwise Specified; HCC‐NOS) [1, 8, 11]. Scirrhous hepatocellular carcinoma shows extensive intratumoral fibrosis [1, 8, 11]. Previously, several studies reported relationships between molecular and pathological features in HCC [3, 14, 15, 16]. Well‐differentiated tumors with cholestasis, macrotrabecular, and pseudoglandular growth patterns (GP) were associated with Catenin‐beta 1 (CTNNB1) gene mutations [14, 17]. The macrotrabecular‐massive GP was associated with tumor protein p53 (TP53) gene inactivation and a worse prognosis [14].

The growing understanding of HCC morphological subtyping and HCC phenotypes holds promise for future targeted therapies and more personalized patient follow‐up. However, its translation into clinical practice will require precise histopathological reporting of all pathomorphological features [16]. For this purpose, the International Collaboration on Cancer Reporting (ICCR) developed a data set for the reporting of HCC, consisting of several items, such as maximal dimension, histological tumor type, histological grade, vascular invasion, margin status, lymph nodes status, and pathological staging according to the TNM 8th edition. Moreover, a few recommended data items, such as satellitosis, macroscopic neoplastic GP, macroscopic tumor rupture, and response to neoadjuvant therapy, may also be reported [18, 19].

This study aimed to analyze the HCC morphological subtypes according to the WHO classification and ICCR data set and their prognostic features in a Dutch HCC patient population of a tertiary referral center.

## 2 Materials and Methods

This retrospective single tertiary center cohort study initially included 408 HCC patients seen at the Maastricht University Medical Center + (MUMC +), Maastricht, the Netherlands, between 2011 and 2021. The study was approved by the Medical Ethics Review Committee of the MUMC +, Maastricht, the Netherlands (METC‐number: 2018‐0703‐A‐10 and 2019‐0977) and adherent to the principles of the Helsinki Declaration [20]. Tissue samples were collected and studied according to the protocol of the Dutch Code of Conduct for Observational Research with Personal Data (2004) and Tissue [21]. Exclusion criteria were no definite diagnosis of HCC, age under 18, diagnosis of fibrolamellar HCC, neo‐adjuvant therapy, and patient's objection against using the material. Finally, 62 HCC patients were included in this study.

### 2.1 Clinic Data and Histopathology

Demographic data on gender, date of birth, and age at primary diagnosis were collected from the patient's medical files. Resection H&E slides were retrieved from the archive of the Department of Pathology and revised according to the current WHO 2019 classification guidelines and the International Collaboration on Cancer Reporting (ICCR) Hepatocellular Carcinoma Histopathology Reporting Guide (2nd edition) by three pathologists (IVS, JCB, AzH) [18, 19]. Data on surgical specimen, tumor characteristics (focality, site, size, number of submitted blocks, satellitosis, histologic type, histologic grade, growth pattern), tumor extension, vascular and perineural invasion, regional LNs, resection margins, and the background liver parenchyma were recorded. The pathologic stage classification according to pTNM, 8th edition was applied to all tumors [22].

**FIGURE 1:**
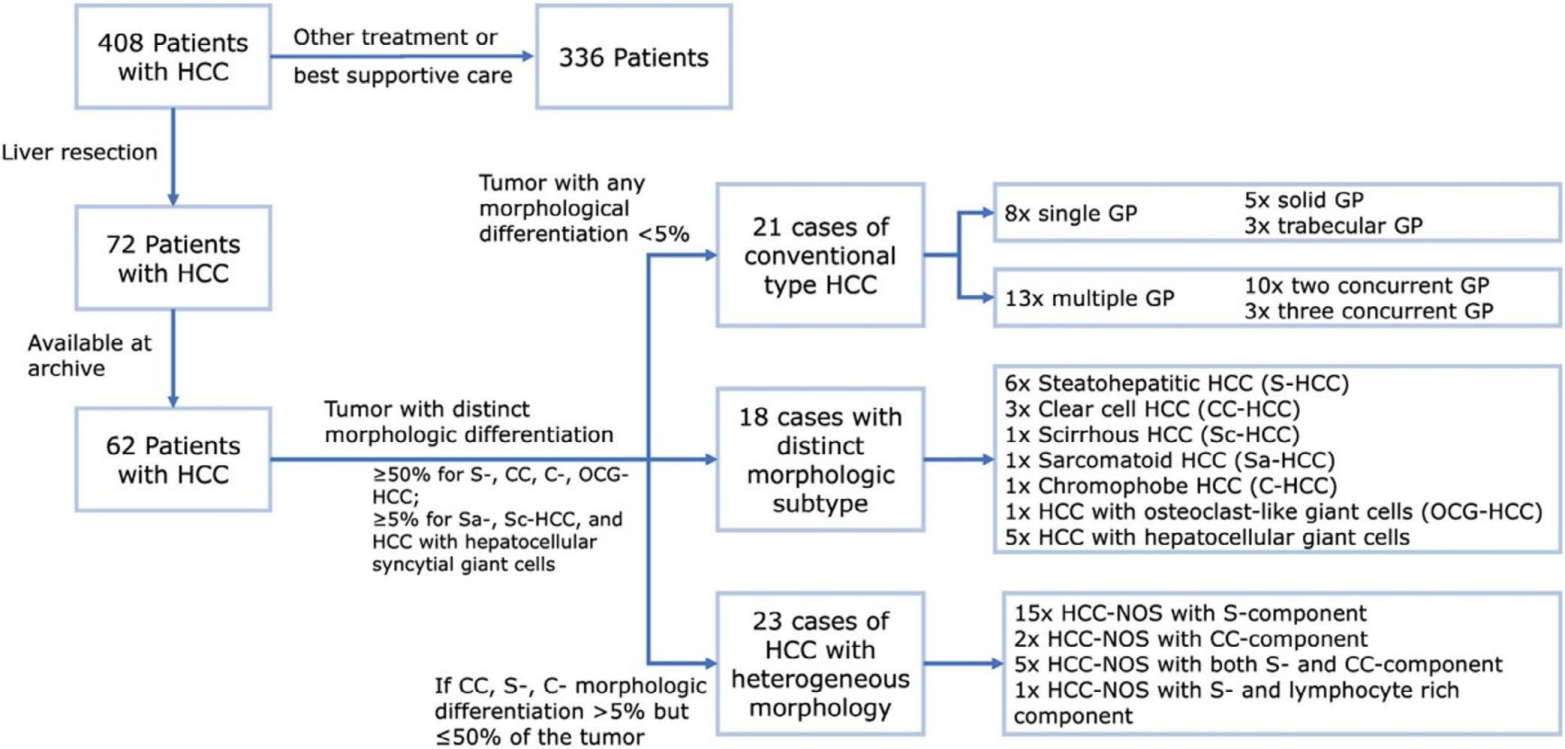
Schematic description of the classification of the cases according to morphologic subtype and growth pattern suggested by the WHO 2019 classification.

HCCs exhibiting a morphologic subtypes were classified accordingly (Figure 1). If a tumor exhibited different morphological pattern, all components were quantified, as a percentage of the whole tumor volume, and these tumors were classified as HCC with heterogeneous morphological patterns (H‐HCC) (Figure 1). The morphological subtypes of HCC were assigned as described previously [1, 11]. Steatohepatitic HCC is defined by its characteristic feature, such as steatosis, cell ballooning, and Mallory‐Denk bodies, in more than 50% of the tumor [11]. Clear cell HCC is defined by presence of clear cell changes in more than 50% of the tumor [1, 11]. The sarcomatoid HCC is defined by spindle cell morphology with immunoreactivity to keratin by immunohistochemistry, confirming their epithelial differentiation, however without mesenchymal component [11]. Chromophobe HCC (C‐HCC) is defined by neoplastic cells with amphophilic/eosinophilic cytoplasm with bland nuclei, in more than 50% of the tumor. Hepatocellular carcinoma with syncytial giant cells shows cells similar to giant cells seen in infantile giant cell hepatitis, in more than 5% of the tumor.

All remaining HCCs were classified as a conventional type HCC (HCC‐NOS) (Figure 1) [19]. In HCC‐NOS, the growth pattern was evaluated as previously reported [11, 19]. Solid growth is defined by broad sheets of tumor cells without visible trabeculae at low to medium magnification (4×, 10×, and 20× magnification); trabecular growth is defined by thin to moderately thick trabeculae seen at low to medium magnification (4×, 10×, and 20× magnification); macrotrabecular growth is defined as cells growing in trabeculae (at least 10 cells in thickness), and finally, pseudoglandular growth is defined as round‐oval hepatocellular structures.

### 2.2 Statistical Analysis

All statistical analysis was performed using IBM SPSS Statistics 27 (IBM Inc., Chicago, IL, USA). Statistical significance was set at *p* < 0.05. To characterize the patient cohort descriptive statistics were used. Survival analysis was done using the Kaplan–Meier (KM) method and the log‐rank test. Spearman's rank correlation was computed to assess the associations among the clinicopathological parameters.

## 3 Results

### 3.1 Clinicopathological Characteristics

Of the total 408 patients, 72 received liver resection. Sixty‐two liver resections were available for revision. The cohort consisted of 22 (35.5%) female patients and 40 (64.5%) male patients. The median age was 67 years [range 28–88 years]. The median overall survival was 46 months [range 1–103 months]. Median tumor size was 7.1 cm [range 0.5–18 cm]. Fifty‐seven (92%) tumors were confined to the liver with free resection margins in 52 (83.9%) cases. The median number of tissue blocks submitted for histopathological analysis was 8 [range 1–26], depending on the tumor size. Tumors with a diameter under 3 cm were always submitted completely. At least one tissue block per cm of tumor diameter was sampled in the larger tumors as well as all macroscopically distinct areas were sampled. 18 (29%) of the tumors exhibited satellitosis. Vascular invasion, defined previously as the presence of tumor cells within the vascular cavities of the portal and hepatic venous systems, was a common feature (*n* = 50, 80.6%) [23]. A positive correlation was found between vascular invasion and tumor diameter (r(df) = 0.356, *p* = 0.005). Perineural growth was infrequent (*n* = 4, 6.4%). Among the TNM stages, stage II was the most frequent (*n* = 43, 69.4%) (Table 1).

**TABLE 1:** Demographic and clinical characteristics of patients with liver resection (*n* = 62).

Variable				*n*	
Age at resection in years, mean (range)				67	28–88
Median survival in months, median (range)				46	1–103
					Percentage (%)
Gender					
Female				22	35.5
Male				40	64.5
Surgical specimen					
Partial hepatectomy				29	46.8
Major hepatectomy (≥ 3 segments)				19	30.6
Minor hepatectomy (< 3 segments)				9	14.5
Other				5	8.1
Tumor characteristics					
*Tumor focality*					
Solitary				57	91.9
Multiple				5	8.1
*Tumor site*					
Right lobe				14	22.6
Left lobe				10	16.1
Segmental location				32	51.6
Other				6	9.7
*Tumor size*					
Greatest dimension in cm, mean (range)				7.1	0.5–18
Neo‐adjuvant therapy				0	0
Satellitosis				18	29
Tumor extension					
Tumor confined to liver				57	92
Tumor involves hepatic vein				4	6.4
Tumor invades adjacent organs				1	1.6
Margins					
Free				52	83.9
Mean distance from margins (mm), mean (range)				4	0.3–20
Involved				10	16.1
Vascular invasion				50	80.6
Microvascular invasion^a^				45	90.0
Macrovascular invasion^b^				5	10.0
Perineural invasion				4	6.4
Regional lymph nodes included in resection					
Lymph nodes containing HCC metastasis				113	17.7
pTNM classification					
m (multiple primary tumors)				5	8.0
*Primary tumor*					
pT1a: solitary tumor ≤ 2 cm (greatest dimension) with or without vascular invasion				4	6.4
pT1b: solitary tumor > 2 cm (greatest dimension) without vascular invasion				7	11.3
pT2: solitary tumor with vascular invasion > 2 cm (greatest dimension) or multiple tumors none > 5 cm (greatest dimension)				45	72.6
pT3: multiple tumors any > 5 cm (greatest dimension)				2	3.2
pT4: tumor(s) involving a major branch of the portal or hepatic vein with direct invasion of adjacent organs (including diaphragm) other than gallbladder or with perforation of visceral peritoneum				4	6.4
*Regional lymph nodes*					
pNX: regional lymph nodes cannot be assessed				51	82.3
pN0: no reginal lymph node metastasis				9	14.5
pN1: regional lymph node metastasis				2	3.2
*Distant metastasis*				3	4.8
Stage					
IA	T1a	N0	M0	3	4.8
IB	T1b	N0	M0	6	9.7
II	T2	N0	M0	43	69.4
IIIA	T3	N0	M0	2	3.2
IIIB	T4	N0	M0	3	4.8
IVA	Any	N1	M0	2	3.2
IVB	Any	Any	M1	3	4.8
Additional pathologic findings (multiple possible)					
None				15	
Fibrosis				16	
Cirrhosis				21	
High‐grade dysplastic nodule				5	
Non‐alcoholic fatty liver disease				27	
Non‐alcoholic steatohepatitis				13	
Iron overload				1	
Chronic hepatitis				2	
Other				1	

^a^ Microvascular invasion is defined as small portal or hepatic vein, or microvascular invasion present microscopically.

^b^ Macrovascular invasion is defined as large portal or hepatic vein invasion present macroscopically.

### 3.2 Histopathology of HCC

#### 3.2.1 HCC NOS

**FIGURE 2:**
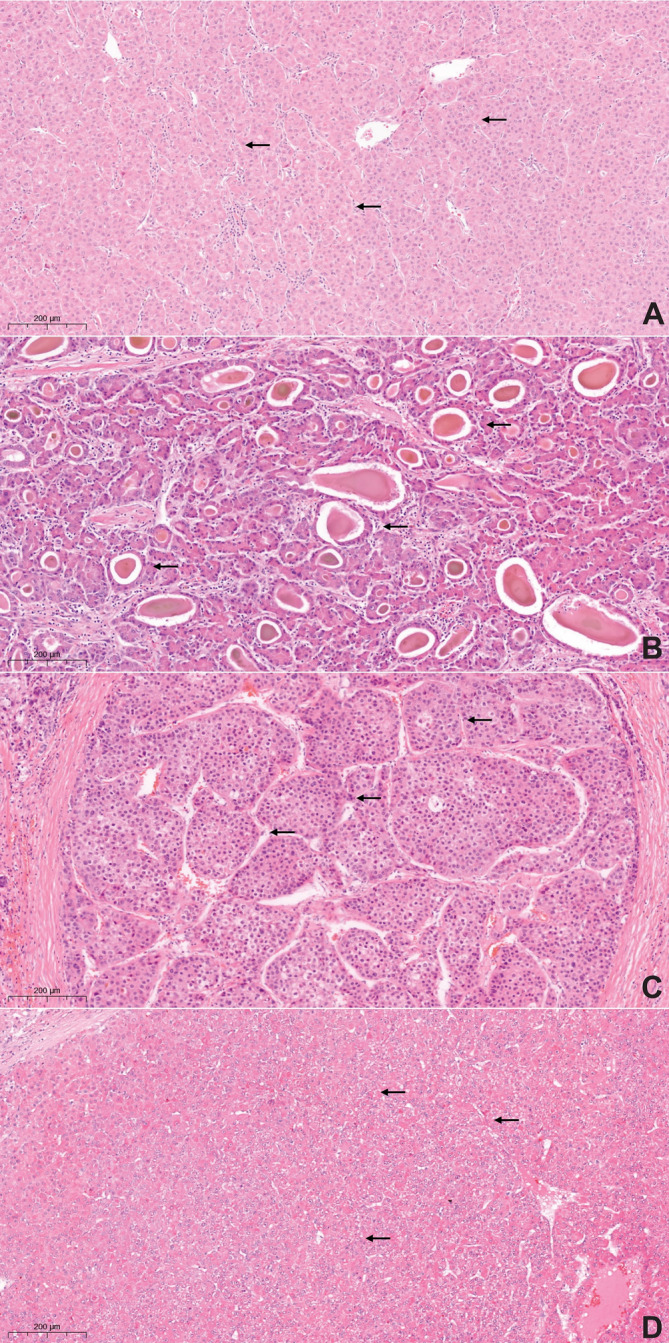
Hepatocellular carcinoma, conventional type (HCC‐NOS) with different growth patterns (hematoxylin–eosin stain, 20× magnification). (A) Trabecular growth pattern. The arrows highlight the thickened trabeculae of polygonal, eosinophilic tumor cells; (B) Pseudoglandular growth pattern. The arrows show dilated acinar structures, lined with a layer of neoplastic cells, often with fluid in the lumen; (C) Macrotrabecular growth pattern. The arrows highlight the large thickened trabeculae, being ≥ 10 cells thick. (D) Solid growth pattern. The arrows show compact growth of the neoplastic cells.

Conventional type HCC (HCC‐NOS) was seen in 21 (33.9%) cases (Tables 2 and 3). This group was characterized by male predominance (80.1%). While eight of these tumors (38.1%) exhibited a single growth pattern (GP), either solid or trabecular (Figure 1, Figure 2), 13 tumors (61.9%) demonstrated multiple GPs, and three tumors (14.3%) even showed three concomitant GPs (Table 3, Figure 1). There was a negative correlation between the vascular invasion and single GP (either solid or trabecular) in HCC‐NOS (r(df) = −0.322, *p* = 0.011). No correlation was found between GPs, tumor diameter and lymph node status.

**TABLE 2:** Clinicopathological data of the different morphological subgroups in our HCC cohort (total number of the cases *n* = 62).

HCC subtype	Number of patients (% of the total cases)	Mean age at diagnosis in years, [range]	Male gender, number (%)	Mean duration of follow‐up in months, [range]	Loss to follow‐up, number, (%)	Outcome (death), number, (%)	Mean serum AFP, kU/L, [range]	Measured mean tumor diameter, cm, [range]	Well differentiated, number, (%)	Stage II, number, (%)	Satellitosis, number, (%)	Vascular invasion, number, (%)	Perineural invasion, number, (%)
HCC NOS	21 (33.9%)	63.2 [45–80]	17 (80.1%)^a^	30 [1–88]	1 (4.8%)	9 (42.9%)	5553.5 [2.1–73 000]	7.48 [0.7–18]	15 (71.4%)	11 (52.8%)^b^	5 (23.8%)	14 (66.7%)	1 (4.8%)
HCC with heterogeneous morphology	23 (37.1%)	66.69[28–88]	12 (52.2%)^a^	26 [1–80]	0 (0%)	11 (47.8%)	1308.2 [2.1–29 000]	7.1 [1.7–15.5]	18 (78.3%)	19 (82.6%)^b^	8 (34.8%)	20 (87%)	1 (4.3%)
Steatohepatic	6 (9.7%)	72.2 [66–75]	5 (83.3%)	18 [6–54]	0 (0%)	1 (16.7%)	8.7 [1.8–29]	5.37 [2.5–8.5]	5 (83.3%)	4 (66.7%)	3 (50%)	5 (83.3%)	0 (0%)
HCC with hepatocellular giant cells	5 (8.0%)	73.2[48–83]	3 (60%)	48 [4–103]	0 (0%)	2 (40%)	6213.7 [3.6–28 740]	8.68 [2.9–17]	3 (60%)	4 (80%)	1 (16.7%)	5 (100%)	1 (20%)
Clear cell	3 (4.8%)	66.33 [63–71]	1 (33.3%)	10 [2–19]	0 (0%)	3 (100%)	49.5 [8.2–75.2]	3.67 [0.5–7]	2 (66.7%)	2 (66.7%)	0 (0%)	2 (66.7%)	0 (0%)
Scirrhous	1 (1.6%)	64	1 (100%)	8	1 (100%)	—	5.5	1.2	0	0 (0%)	0 (0%)	1 (100%)	0 (0%)
Chromophobe	1 (1.6%)	75	1 (100%)	30	0 (0%)	1 (100%)	6.8	7	0	1 (100%)	0 (0%)	1 (100%)	0 (0%)
Sarcomatoid	1 (1.6%)	63	0 (0%)	4	0 (0%)	1 (100%)	—	13	0	1 (100%)	1 (100%)	1 (100%)	0 (0%)
HCC with osteoclast‐like giant cells	1 (1.6%)	77	1 (100%)	6	0 (0%)	0 (0%)	4.7	12.2	0	1 (100%)	1 (100%)	1 (100%)	0 (0%)

*Note:* Morphologic classification according to [14].

Abbreviations: HCC, hepatocellular carcinoma; NOS, not otherwise specified.

^a^ *p* = 0.41, χ^2^ = 4.172, Chi‐square test.

^b^ *p* = 0.03, χ^2^ = 4.725, Chi‐square test.

**TABLE 3:** Clinicopathological characteristics of HCC NOS group (*n* = 21) depending on the growth pattern.

Growth pattern HCC NOS (*n* = 21)	Number	Mean age at diagnosis in years (range)	Male gender in number (%)	Mean duration of follow‐up in months (range)	Loss to follow‐up, number (%)	Outcome (death), number (%)	Mean serum AFP kU/L, (range)	Mean diameter in cm (range)	Well differentiated, number (%)	Stage II, number (%)	Satellitosis, number (%)	Vascular invasion, number (%)	Perineural invasion, number (%)
Solitary growth pattern													
Solid	5	65.4 y (55–77)	3 (60%)	42 m (5–88)	0 (0%)	1 (20%)	74.12 (2.3–357.4)	7.96 cm (1.1–14.5)	5 (100%)	4 (80%)	1 (20%)	3 (60%)	0 (0%)
Trabecular	3	59.66 y (49–68)	2 (66.66%)	27 m(11–36)	0 (0%)	1 (33.33%)	438.15 (4.3–872)	8.16 cm(1.5–18)	2 (66.66%)	1 (33.33%)	1 (33.33%)	1 (33.33%)	0 (0%)
Multiple growth patterns													
Two trabecular‐pseudoglandular	4	69 y (61–80)	4 (100%)	13 m (1–38)	0 (0%)	1 (25%)	105 325 (4–402)	7 cm (2.2–14.5)	3 (75%)	3 (75%)0	1 (25%)	3 (75%)	0 (0%)
Solid‐trabecular	2	71 y (65–77)	2 (100%)	42 m (3–81)	0 (0%)	1 (50%)	3701.55 (2.1–7401)	2.4 cm (1.8–3.0)	1 (50%)	(0%)	0 (0%)	2 (100%)	1 (50%)
Solid‐macrotrabecular	2	59 y (55–63)	2 (100%)	24 m (6–41)	0 (0%)	1 (50%)	1165.15 (4.3–23 326)	6.25 cm (1.5–11)	2 (100%)	1 (50%)	1 (50%)	1 (50%)	0 (0%)
Solid‐pseudoglandular	2	66.5 y (60–73)	2 (100%)	21 m (21)	1 (50%)	2 (100%)	36 503 (6.2–73 000)	8.85 cm (0.7–17)	0 (0%)	0 (0%)	0 (0%)	1 (50%)	0 (0%)
Three	3	50.66 y (45–57)	2 (66.66%)	32 m (4–55)	0 (0%)	2 (66.66%)	54.15 (53.3–55)	10 cm (6.5–12)	2 (66.66%)	2 (66.66%)	1 (33.33)	3 (100%)	0 (0%)

### 3.3 Morphologic Subtypes

**FIGURE 3:**
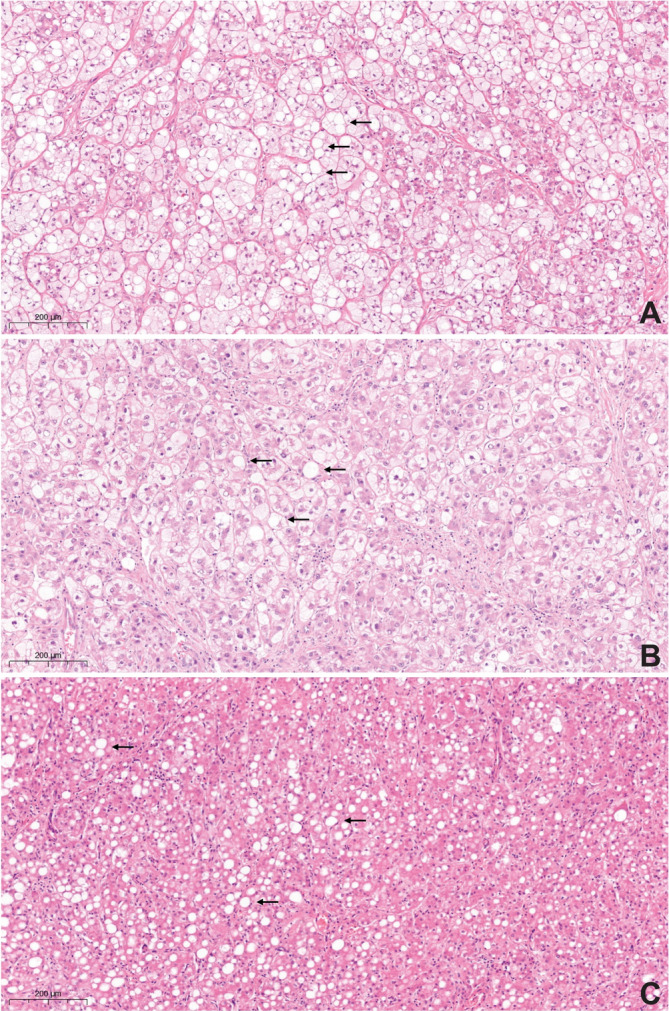
Steatotic hepatocellular carcinoma (hematoxylin–eosin stain, 20× magnification). In all three demonstrated cases (A–C), neoplastic cells showed macrovesicular steatosis and a different degree of ballooning, highlighted by arrows.

**FIGURE 4:**
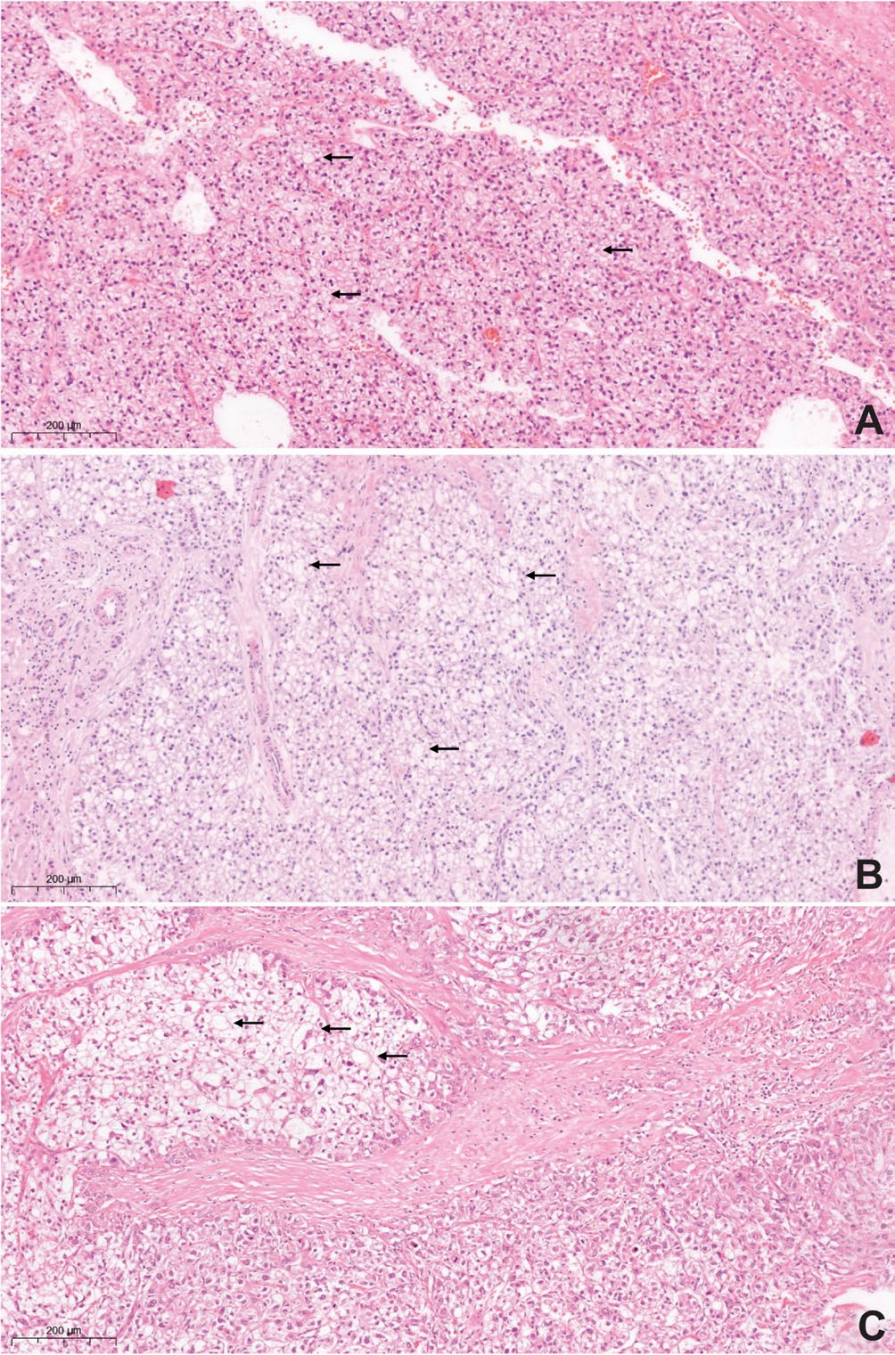
Clear cell hepatocellular carcinoma (hematoxylin–eosin stain, 20× magnification). Neoplastic cells showed clear cytoplasm in all three demonstrated cases (A–C), indicated with arrows.

**FIGURE 5:**
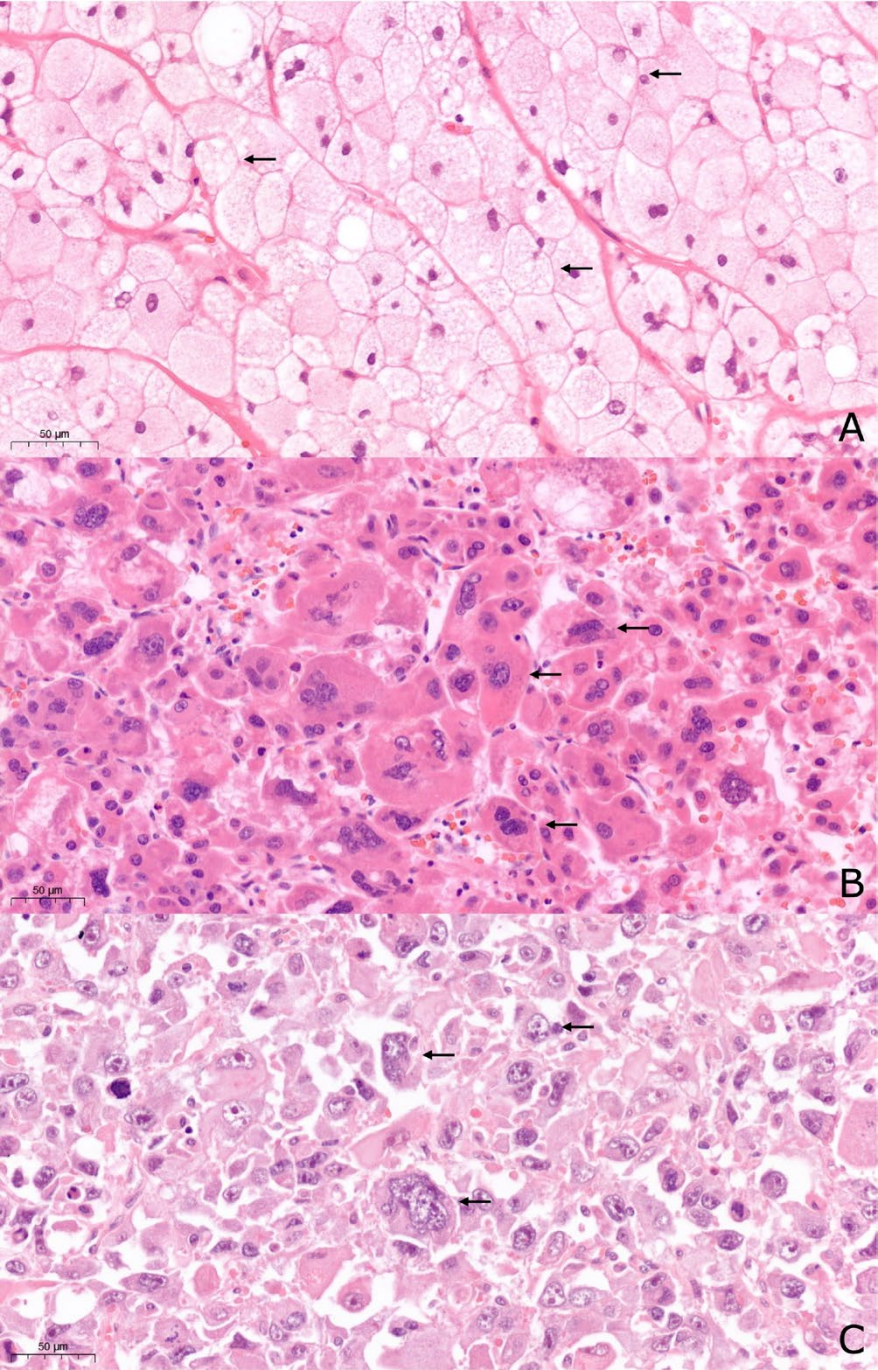
Rare subtypes of hepatocellular carcinoma (hematoxylin–eosin stain, 20× magnification). (A) Chromophobe HCC. The arrows show neoplastic cells with smooth clear cytoplasm with distinct cell membranes; (B) HCC with osteoclast‐like giant cells. The arrows indicate the large multinucleated cells with eosinophilic cytoplasm. (C) HCC with syncytial giant cells. The arrows show intratumoral giant cells with atypical nuclei.

Eighteen tumors (29.0%) displayed a distinct morphologic subtype of HCC (Table 2, Figures 3 and 4). This group compromised six steatohepatitic HCCs (S‐HCC), three clear cell HCCs (CC‐HCC), one scirrhous HCC, and one sarcomatoid HCC. Three provisional morphologic subtypes were found: one chromophobe HCC (C‐HCC), one HCC with osteoclast‐like giant cells, and five HCC with syncytial giant cells (Table 2, Figure 5). The osteoclast‐like giant cells were observed in about 30% of the tumor, these were positive for immunohistochemistry with CD68 while negative for HepPar1 [24]. The five HCCs with osteoclast‐like giant cells included well to moderately differentiated HCC‐NOS with at least 5% of hepatocellular syncytial giant cells, confirmed by immunohistochemistry with HepPar1. There was no correlation between the different morphologic subtypes and the clinicopathological characteristics (vascular invasion, tumor diameter, lymph node status).

### 3.4 HCC With Heterogeneous Morphology

Twenty‐three cases (37.1%) displayed a heterogeneous morphology, meaning a combination of HCC‐NOS with another morphologic subtype (Figure 1). Most cases (15 tumors) showed a combination of HCC‐NOS and a steatohepatitic component, which varied from 5% to 45% of the total tumor volume. Two cases showed a clear cell component in about 30% of the total tumor volume. One tumor revealed a prominent lymphocytic infiltrate in about 30% of the tumor, suggesting lymphocyte‐rich HCC component (Table 2). Five cases exhibited three morphologic subtypes: HCC‐NOS with different proportions of concurrent steatohepatitic and clear‐cells. Compared with HCC‐NOS, more female patients demonstrated HCC with heterogeneous morphology (Table 2, *p* = 0.41, χ^2^ = 4.172, Chi‐square test) and the number of Stage II tumors was higher in this group (Table 2, *p* = 0.03, χ^2^ = 4.25, Chi‐square test).

### 3.5 Survival Analysis

Except for maximal tumor diameter, which was significantly associated with survival (log‐rank 56.618, *p* = 0.043), overall survival was independent of age, gender, satellitosis, vascular or perineural invasion, morphologic subtype, GP, histologic grade, tumor stage, and background liver cirrhosis. Moreover, no differences in survival were found between groups of HCC‐NOS and HCC with heterogeneous morphology (log‐rank = 0.39, *p* = 0.6).

## 4 Discussion

HCC is the most common liver cancer, characterized by histomorphological heterogeneity. Many histopathological features, such as vascular invasion and tumor diameter, are widely accepted as prognostic factors. Nowadays, morphologic subtypes are thought to be promising for improved prediction of patient outcome. This retrospective study carried out at a tertiary referral center evaluated the histomorphological parameters of 62 HCC resection specimens according to the new WHO 2019 classification (including the four GPs, 12 distinct morphologic and six provisional subtypes of HCC) [10]. Furthermore, the histological parameters were evaluated according to the International Collaboration on Cancer Reporting (ICCR) data set for the reporting of hepatocellular carcinoma [18].

The new WHO 2019 classification describes that about 35% of HCCs can be assigned one of the 12 morphological subtypes or one of the six provisional morphologic subtypes [1, 3, 8, 11, 12, 13, 25, 26, 27, 28]. In our cohort, 29% of the analyzed HCCs were assigned a specific morphological subtype. These were in decreasing frequency: steatohepatic HCC, HCC with hepatocelullar giant cells, clear cell HCC and chromophobe HCC, sarcomatoid HCC, and HCC with osteoclast‐like giant cells (OCG‐HCC). Only a few case‐reports on OGC‐HCC were reported [24, 29]. This tumor is characterized by rather aggressive behavior, shows a normal AFP level, and has a poor prognosis [24, 30]. Another rarely reported tumor was HCC with hepatocellular syncytial giant cells, of which we identified five tumors in our cohort. In this subtype of HCC, numerous syncytial multinucleated giant cells, similar to those seen in infantile giant cell hepatitis, are observed [1, 11, 17, 25, 28, 30]. Despite the presence of the multinucleated cells, the tumors are usually well to moderately differentiated, as also seen in our specimens. The primary differential diagnosis is OGC‐HCC; therefore, immunohistochemical analysis (Hepar1 and CD68) is required to confirm the diagnosis. Prior to this study, only a limited number of such cases have been cited. Therefore, this tumor's characteristics, biological behavior and prognosis are largely unknown [30].

Furthermore, 21 tumors (33.9%) were classified as conventional type HCC (HCC‐NOS), most commonly exhibiting two concurrent growth patterns (GP) (*n* = 10). In order of decreasing frequency, we observed two concurrent GPs, single solid, single trabecular, and mixed three concurrent GPs. Pseudoglandular and macrotrabecular growth occurred only in conjunction with solid or trabecular growth. Generally, the results of our study are in line with previous studies evaluating GPs in HCCs [1, 11]. However, frequencies of GPs vary greatly depending on the cut‐off values and specimen type (resection versus biopsy) [25]. Our study was performed on resection specimens with a high number of submitted tissue blocks (the median number of tissue blocks was 8 [range 1–26], depending on the tumor diameter). Tumors under 3 cm were always submitted completely. At least one tissue block per cm of tumor diameter and all macroscopically distinct areas were sampled. This approach is in line with the previously mentioned data set for reporting HCC. The ICCR recommends examining a minimum of three tumor blocks, with sampling of all macroscopically distinctive areas. Examination of the whole cross‐section has been suggested if the tumor is smaller than 2 cm with an additional section for each centimeter for larger tumors [18]. Additionally, morphologic subtypes can be recommended for reporting. In HCC with heterogeneous morphology, we recommend to report the percentages of the morphological components.

Our study showed that the majority of HCCs in this cohort display heterogeneous morphological differentiation, compromising the combination of HCC‐NOS (HCC‐NOS) with another morphologic subtype (with either a steatohepatitic component, clear cell component, combined steatohepatitic and clear cell component, or lymphocyte‐rich component). Except for proportion of female patients and number of stage II tumors, comparison of HCC‐NOS and HCC with heterogeneous morphology did not show significant differences regarding the main clinicopathological characteristics and overall survival. This finding suggests that surgical treatment is an effective strategy for HCC, irrespective of morphological subtypes. Thus, surgery could remain the first‐line therapeutic approach for appropriate groups of patients, given its potential to achieve favorable outcomes. Further studies on HCC with heterogeneous morphology are necessary to understand their biological behavior and the implication on tumor prognosis.

Description of vascular invasion and perineural invasion in the pathology report is recommended by the ICCR because of the correlation with adverse tumor biology and poor prognosis. According to the literature, the incidence of vascular invasion varies between 15% and 57.1%, depending on specimen sampling and reporting of this diagnostic criterion. In our cohort, vascular invasion was identified in about 81% of cases and positively correlated with the tumor diameter. It aligns with previously published data: larger tumors have a higher incidence of vascular invasion and intrahepatic metastasis, which are frequently associated with a poor prognosis [23]. Perineural invasion was identified rarely. The clinical significance of perineural invasion is not yet fully understood; however, a potential role was suggested in bone metastasis [31].

The main limitation of this retrospective study is the relatively limited number of patients, restricting the ability to draw definitive conclusions regarding the association between morphologic parameters and survival. Furthermore, the demography of our patient cohort, which primarily consists of Caucasian patients, may limit the extrapolation of our findings to populations with different ethnic backgrounds. However, since most data that is currently available is derived from Asian cohorts, this study contributes to a more comprehensive understanding of the subject of HCC morphology.

Combining data from various centers and cohorts is essential for better understanding of the clinical relevance of HCC subtyping and could potentially aid in the development of a predictive model that also integrates the morphological subtype of HCC. In order to achieve this goal, it would be advantageous of other research groups re‐evaluated their histopathological specimens using the updated WHO 2019 and ICCR guidelines.

In conclusion, despite the limited number of patients in our cohort, our results showed distinct morphologic heterogeneity in resection specimens of HCC. We recommend multiple preoperative biopsies of different tumor regions to ensure adequate sampling, thereby addressing the morphologic heterogeneity. For the evaluation of surgical samples the ICCR guidelines for the HCC reporting should be endorsed [18]. Thus, awareness of HCC intratumoral heterogeneity and subsequent adaptation of sampling protocols will improve the correct classification of HCCs, with potential significant implications for clinical outcomes and treatment strategies.

## Author Contributions

**C. H. A. Saler:** conceptualization (equal), data curation (equal), formal analysis (equal), funding acquisition (equal), investigation (equal), methodology (equal), project administration (equal), resources (equal), software (equal), supervision (equal), validation (equal), visualization (equal), writing – original draft (equal), writing – review and editing (equal). **S. Shuai:** formal analysis (equal), project administration (equal), writing – review and editing (equal). **J. C. Beckervordersandforth:** conceptualization (equal), formal analysis (equal), funding acquisition (equal), investigation (equal), methodology (equal), validation (equal), visualization (equal), writing – review and editing (equal). **D. Rennspiess:** investigation (equal), methodology (equal), resources (equal), writing – review and editing (equal). **G. Roemen:** formal analysis (equal), resources (equal), visualization (equal), writing – review and editing (equal). **T. Gevers:** formal analysis (equal), investigation (equal), methodology (equal), resources (equal), visualization (equal), writing – review and editing (equal). **M. C. F. Stoehr‐Kleinegris:** investigation (equal), methodology (equal), project administration (equal), visualization (equal), writing – review and editing (equal). **S. A. W. Bouwense:** data curation (equal), funding acquisition (equal), methodology (equal), software (equal), visualization (equal), writing – review and editing (equal). **M. J. L. Dewulf:** formal analysis (equal), investigation (equal), methodology (equal), visualization (equal), writing – review and editing (equal). **M. M. E. Coolsen:** conceptualization (equal), data curation (equal), investigation (equal), methodology (equal), resources (equal), writing – review and editing (equal). **M. H. A. Bemelmans:** investigation (equal), methodology (equal), project administration (equal), validation (equal), writing – review and editing (equal). **S. W. Olde Damink:** formal analysis (equal), investigation (equal), methodology (equal), project administration (equal), validation (equal), writing – review and editing (equal). **V. Winnepenninckx:** investigation (equal), methodology (equal), project administration (equal), resources (equal), validation (equal), writing – review and editing (equal). **A. zur Hausen:** conceptualization (equal), data curation (equal), formal analysis (equal), funding acquisition (equal), investigation (equal), methodology (equal), project administration (equal), resources (equal), software (equal), supervision (equal), validation (equal), visualization (equal), writing – original draft (equal), writing – review and editing (equal). **M. Kramer:** conceptualization (equal), data curation (equal), formal analysis (equal), funding acquisition (equal), investigation (equal), methodology (equal), project administration (equal), resources (equal), software (equal), supervision (equal), validation (equal), visualization (equal), writing – original draft (equal), writing – review and editing (equal). **I. V. Samarska:** conceptualization (equal), data curation (equal), formal analysis (equal), funding acquisition (equal), investigation (equal), methodology (equal), project administration (equal), resources (equal), software (equal), supervision (equal), validation (equal), visualization (equal), writing – original draft (equal), writing – review and editing (equal).

## Conflicts of Interest

The authors declare no conflicts of interest.

## Data Availability

The data that support the findings of this study are available on request from the corresponding author.
